# Supplementary material for: HRI coordinates translation necessary for protein homeostasis and mitochondrial function in erythropoiesis
Source: eLife. 2019 Apr 29;8:e46976. doi: 10.7554/eLife.46976 (PMC6533081; doi:10.7554/eLife.46976)
Supplement: Transparent reporting form [file elife-46976-transrepform.docx]

***eLife’s* transparent reporting form**

We encourage authors to provide detailed information *within their submission* to facilitate the interpretation and replication of experiments. Authors can upload supporting documentation to indicate the use of appropriate reporting guidelines for health-related research (see [EQUATOR Network](http://www.equator-network.org/%20)), life science research (see the [BioSharing Information Resource](https://biosharing.org/)), or the [ARRIVE guidelines](http://www.plosbiology.org/article/info:doi/10.1371/journal.pbio.1000412) for reporting work involving animal research. Where applicable, authors should refer to any relevant reporting standards documents in this form.

If you have any questions, please consult our Journal Policies and/or contact us: [editorial@elifesciences.org](mailto:editorial@elifesciences.org).

**Sample-size estimation**

- You should state whether an appropriate sample size was computed when the study was being designed
- You should state the statistical method of sample size computation and any required assumptions
- If no explicit power analysis was used, you should describe how you decided what sample (replicate) size (number) to use

Please outline where this information can be found within the submission (e.g., sections or figure legends), or explain why this information doesn’t apply to your submission:

Our study is not a population-based research. The erythroid phenotypes of HRI null mice is very pronounced with 100% penetration as shown in our previous publications. In the designs of present study, we generally performed 3 or more biological replicas.

**Replicates**

- You should report how often each experiment was performed
- You should include a definition of biological versus technical replication
- The data obtained should be provided and sufficient information should be provided to indicate the number of independent biological and/or technical replicates
- If you encountered any outliers, you should describe how these were handled
- Criteria for exclusion/inclusion of data should be clearly stated
- High-throughput sequence data should be uploaded before submission, with a private link for reviewers provided (these are available from both GEO and ArrayExpress)

Please outline where this information can be found within the submission (e.g., sections or figure legends), or explain why this information doesn’t apply to your submission:

We defined technical replication as performing duplicate assays using the same starting material, while biological replication is defined as performing assays using different starting biological samples. We used four different conditions, wild type and HRI null E14.5 fetal livers under iron sufficient and deficient conditions to investigate the roles of HRI and iron on the gene expression of primary basophilic erythroblasts. In order to have sufficient EBs for Ribo-seq library, fetal livers from embryos of the same mother were pooled and then sorted for basophilic erythroblasts as one biological replica. Two replicas of 5 million cells each and three biological replicas of 1 million cells each were obtained from three separate mothers and were used for successful preparations of Ribo-seq and mRNA-seq libraries, respectively. The third replica of Ribo-seq using 3 million cells was unsuccessful likely due to lower cell numbers. This information is relevant to results derived from data sets of Ribo-seq and mRNA-seq presented in the manuscript and can be found in Methods.

Five technical replicas were performed for each condition in each seahorse assay (Figure 4A). Totally, four separate assays were performed and 5-8 biological replicas for each condition were obtained finally (Figure 4B).

In the *ex vivo* differentiation study shown in Figure 5C-D, numbers of fetal livers used for each genotypes were n=6 for *Wt* and *Hri^-/-^*; n=4 for *AA* and n=3 for *Atf4^-/-^*. This information can be found in the legend of Figure 5.

qPCR results were obtained using 3 technical replicas, and were repeated three times with samples of separate fetal livers. This information can be found in the figure legends of Figure supplementary 5-6. For qPCR results in Figure supplementary 4, three biological replicas were used. For western blot analysis and FACS, results of a representative experiment was shown and has been repeated in three separate experiments.

In *Grb10* knockdown experiments (Figure 7), DNA sequences of eight shRNA oligonucleotides targeting different regions of *Grb10* mRNA. This information can be found in the text under the section of *Grb10* results and Methods. Three biological replicas for GFP control, shRNA_G3 and shRNA_G7 were included. Representative cytospin results of *Grb10* knockdown experiments were shown.

**Statistical reporting**

- Statistical analysis methods should be described and justified
- Raw data should be presented in figures whenever informative to do so (typically when N per group is less than 10)
- For each experiment, you should identify the statistical tests used, exact values of N, definitions of center, methods of multiple test correction, and dispersion and precision measures (e.g., mean, median, SD, SEM, confidence intervals; and, for the major substantive results, a measure of effect size (e.g., Pearson's r, Cohen's d)
- Report exact p-values wherever possible alongside the summary statistics and 95% confidence intervals. These should be reported for all key questions and not only when the p-value is less than 0.05.

Please outline where this information can be found within the submission (e.g., sections or figure legends), or explain why this information doesn’t apply to your submission:

Independent t test (two-tailed) was used to analyze the experimental data. Pearson correlation analysis was performed to determine the correlation coefficient. Data were presented in mean ± SE. p < 0.05 was considered statistically significant. This information can be found in Methods and figure legend.

(For large datasets, or papers with a very large number of statistical tests, you may upload a single table file with tests, Ns, etc., with reference to sections in the manuscript.)

**Group allocation**

- Indicate how samples were allocated into experimental groups (in the case of clinical studies, please specify allocation to treatment method); if randomization was used, please also state if restricted randomization was applied
- Indicate if masking was used during group allocation, data collection and/or data analysis

Please outline where this information can be found within the submission (e.g., sections or figure legends), or explain why this information doesn’t apply to your submission:

**Additional data files (“source data”)**

- We encourage you to upload relevant additional data files, such as numerical data that are represented as a graph in a figure, or as a summary table
- Where provided, these should be in the most useful format, and they can be uploaded as “Source data” files linked to a main figure or table
- Include model definition files including the full list of parameters used
- Include code used for data analysis (e.g., R, MatLab)
- Avoid stating that data files are “available upon request”

Please indicate the figures or tables for which source data files have been provided:

All sequencing data have been deposited in GEO under accession codes GSE119365. This information can be found in Methods.
